# The cherry 6+9K SNP array: a cost-effective improvement to the cherry 6K SNP array for genetic studies

**DOI:** 10.1038/s41598-020-64438-x

**Published:** 2020-05-06

**Authors:** Stijn Vanderzande, Ping Zheng, Lichun Cai, Goran Barac, Ksenija Gasic, Dorrie Main, Amy Iezzoni, Cameron Peace

**Affiliations:** 1 Department of Horticulture, Washington State University, Pullman, WA USA; 2 Department of Horticulture, Michigan State University, East Lansing, MI USA; 3 Department of Fruit Growing, Viticulture, Horticulture and Landscape Architecture, University of Novi Sad, Novi Sad, Serbia; 4 Department of Plant and Environmental Sciences, Clemson University, Clemson, SC USA

**Keywords:** Agricultural genetics, Genetic markers, Genomics, Genotype, Haplotypes, Plant breeding, Plant genetics

## Abstract

Cherry breeding and genetic studies can benefit from genome-wide genetic marker assays. Currently, a 6K SNP array enables genome scans in cherry; however, only a third of these SNPs are informative, with low coverage in many genomic regions. Adding previously detected SNPs to this array could provide a cost-efficient upgrade with increased genomic coverage across the 670 cM/352.9 Mb cherry whole genome sequence. For sweet cherry, new SNPs were chosen following a focal point strategy, grouping six to eight SNPs within 10-kb windows with an average of 0.6 cM (627 kb) between focal points. Additional SNPs were chosen to represent important regions. Sweet cherry, the *fruticosa* subgenome of sour cherry, and cherry organellar genomes were targeted with 6942, 2020, and 38 new SNPs, respectively. The +9K add-on provided 2128, 1091, and 70 new reliable, polymorphic SNPs for sweet cherry and the *avium* and the *fruticosa* subgenomes of sour cherry, respectively. For sweet cherry, 1241 reliable polymorphic SNPs formed 237 informative focal points, with another 2504 SNPs in-between. The +9K SNPs increased genetic resolution and genome coverage of the original cherry SNP array and will help increase understanding of the genetic control of key traits and relationships among individuals in cherry.

## Introduction

Genome scan capability has value for cherry breeding and advancing our understanding of crop evolution, diversity, and the genetic control of valuable traits. Sweet cherry (*Prunus avium*) and sour cherry (*P. cerasus*) are two economically important cherry crops that are greatly appreciated for their high-quality fruit. Sweet cherry is diploid (2n = 2×= 16), whereas sour cherry is tetraploid (4n = 2×= 32) that originated through hybridization of sweet cherry with tetraploid ground cherry (*P. fruticosa*)^1^. This hybridization resulted in the presence of two subgenomes in sour cherry: the *avium* subgenome and the *fruticosa* subgenome. Both sweet and sour cherry are clonally propagated crops whose genetic improvement occurs through breeding. As with other tree fruit crops, cherry breeding is limited by its long juvenility period (three to five years) and the limited number of seedlings that produce fruit which meet producer and consumer standards. A better understanding of the genetic architecture of key horticultural traits can help breeders, geneticists, and allied scientist improve breeding efficiency.

A single nucleotide polymorphism (SNP) array was created to improve understanding of cherry genetics. The reported RosBREED cherry 6K SNP array uses the Illumina Infinium technology and encompasses 4212 SNPs targeting the sweet cherry genome and 1482 SNPs targeting the sour cherry genome^2^. Of the SNPs targeting the sour cherry genome, 752 target the *avium* subgenome and 730 target the *fruticosa* subgenome. The cherry 6K SNP array has been used in sweet cherry to deduce pedigree and parentage^3^, create high-density linkage maps^4,5^, identify quantitative trait loci (QTL) and candidate genes controlling important horticultural traits^6,7^, dissect the genetic structure and inheritance of alleles at a QTL hotspot^8^, estimate heritability and genomic breeding values of industry-prioritized traits^9,10^, determine genetic diversity, linkage disequilibrium, and population structure of a germplasm collection and inform development of a core colleciton^11^, and identify patterns of domestication^12^. In sour cherry, the cherry 6K SNP array has been used to identify QTLs and associated alleles for bloom date^13^.

Despite the 6K SNP array’s utility, the number and proportion of informative SNPs was low compared to other arrays for rosaceous crops^2,14–16^. Only 1825 SNPs were polymorphic for 269 sweet cherry accessions and 2058 SNPs were polymorphic for 330 sour cherry accessions^2^. For U.S. sweet cherry breeding-relevant germplasm, the number of reliable polymorphic SNPs was later reduced to 1617 through an extensive curation workflow^17^. Within single sweet cherry cultivars, the number of observed heterozygous SNPs ranged between 313 and 634^17^, which matches the estimation by Peace et al^2^. that 400–700 SNPs would be heterozygous for any given cultivar. This low number of heterozygous SNPs has led to genetic maps with large gaps ranging between 11 and 51 cM for regions not expected to be homozygous^4,5^. A low sequencing depth used to identify polymorphisms has been proposed as one of the reasons for the high monomorphism rate of the cherry 6K SNP array. Additionally, only 314 of the polymorphic sour cherry SNPs targeted the *fruticosa* subgenome.

The utility of SNPs can be increased by grouping them together into multi-marker loci. Unlike bi-allelic single SNPs, these multi-marker loci will usually be multi-allelic. These marker groups reduce computation time and power needed to analyze the data as well as facilitate manual interpretation of the obtained results^18^. This strategy has already been used for the apple 8K and apple 20K SNP arrays^14,16^. For these arrays, SNPs were targeted to be located within ±50 kb (apple 8K SNP array) or ±5 kb (apple 20K SNP array) from focal points that were spread evenly throughout the genome. SNPs near these focal points could then be grouped together to create multi-marker loci. Unfortunately, such utility is currently not available for cherry.

Genotyping-by-Sequencing (GBS) has been proposed to improve the saturation of genetic maps. Using GBS, Guajardo *et al*.^19^ identified 8476 high-quality SNPs in sweet cherry. However, the final number of mapped SNPs ranged between 462 and 985 with the largest gaps on a chromosome ranging between 5.3 and 29.1 cM. In addition, regions covered by GBS might not be the same between different studies, making comparisons between studies harder. Only 16 SNPs mapped by Guajardo *et al*.^19^ were common with the cherry 6K SNP array and thus the GBS study identified a large number of additional SNPs that could be targeted by new genetic tools. Similarly, a GBS study of the population structure and genotypic and phenotypic variability in the natural populations of *P. fruticosa*^20^ provided many SNPs that could be a useful source of SNPs for the *fruticosa* subgenome of sour cherry.

Our goal was to improve the genome coverage and utility of the original cherry 6K SNP array in a cost-effective way. We aimed to add 9000 SNPs to the original array to improve genome coverage while improving utility of the array by adopting a focal point approach previously used for development of the apple 8K and 20K SNP arrays^16^. Cost-effective development of the “+9K add-on” was targeted by using SNPs identified in previous studies that avoided the need for new sequence data.

## Results

### Available SNPs and SNP choice

**Figure 1:**
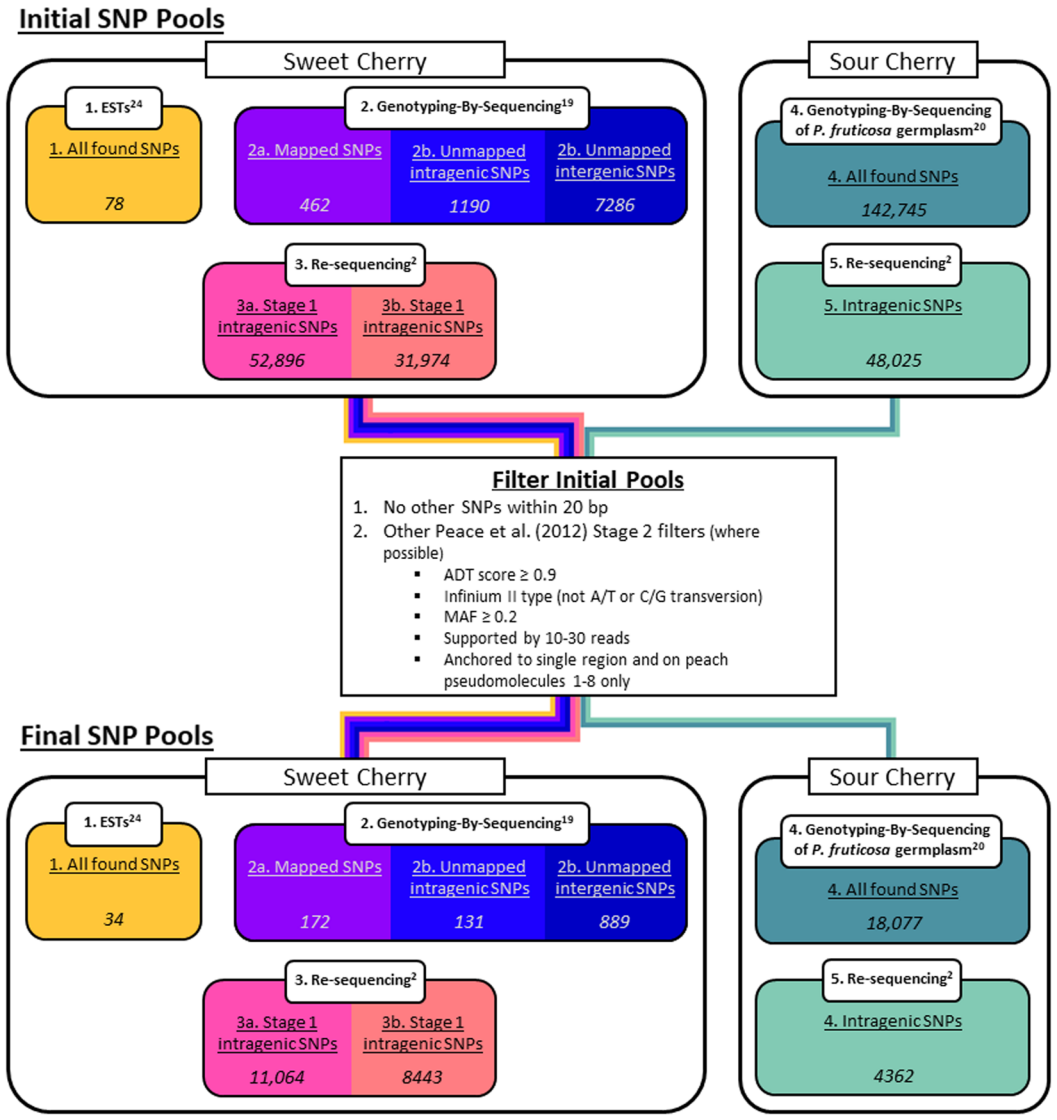
Creation of SNP pools to choose the 9000 SNPs to be added to the original cherry 6K SNP array. For sweet cherry, SNPs were chosen from three sources: a pool of EST SNPs^24^, SNPs identified using GBS on a single seedling populations^19^, and SNPs identified from re-sequencing 16 sweet cherry individuals during development of the original cherry 6K SNP array^2^. For sour cherry, SNPs were chosen from two sources: a pool of SNPs identified through GBS on 32 *P*. *fruticosa* individuals^20^ and a pool of SNPs obtained from re-sequencing eight sour cherry individuals during development of the original cherry 6K SNP array^2^.

A total of 93,886 SNPs were initially available for sweet cherry from existing sequencing sources (Fig. 1, Table 1). Approximately 90% of these SNPs (84,870) were identified from resequencing data used to create the original cherry 6K SNP array. Most of the other SNPs (9.5%; 8938 SNPs) originated from a GBS study in sweet cherry and 78 were from ESTs. Various filter parameters reduced the number of available sweet cherry SNPs for the +9K add-on to 20,733 (22% of initially available SNPs). For the *fruticosa* subgenome of sour cherry, 190,770 SNPs were initially available: 48,025 intragenic SNPs identified during development of the original SNP array and 142,745 SNPs identified through GBS on *P. fruticosa* individuals (Fig. 1, Table 1). This number of *fruticosa* SNPs was reduced to 22,439 (16%) by applying various filter parameters.

**Table 1:** Summary of SNPs available per source at each stage of development of the +9K add-on for the cherry 6K SNP array.

**SNP source**	Initially available SNPs	Stage 2 filters	Chosen for the add-on	On the add-on
***Sweet cherry***	***93,886***	***20,733***	***6942***	***6092***
Source 1 (EST)	78	34	68 (34 ×2)	58
Source 2 (GBS)	8938	1192	691	582
*Source 2a (mapped)*	462	172	172	146
*Source 2b (unmapped intragenic)*	1190	131	61	54
*Source 2c (unmapped intergenic)*	7286	889	458	382
Source 3 (6K array development)	84,870	19,507	6183	5452
*Source 3a (intragenic)*	*52,896*	*11,064*	3783	3331
*Source 3b (intergenic)*	*31,974*	8443	2400	2121
***Sour cherry***	***190,770***	***22,439***	***2020***	***1741***
Source 4 (*P. fruticosa* GBS)	142,745	18,077	1942	1673
Source 5 (6K array development)	48,025	4362	78	68
***Organelle***	***19***	***19***	***38***	***30***
Mitochondrion	3	3	6 (3 ×2)	3
Chloroplast	16	16	32 (16 ×2)	27
**Total**	**284,675**	**43,172**	**9000**	**7863**

**Figure 2:**
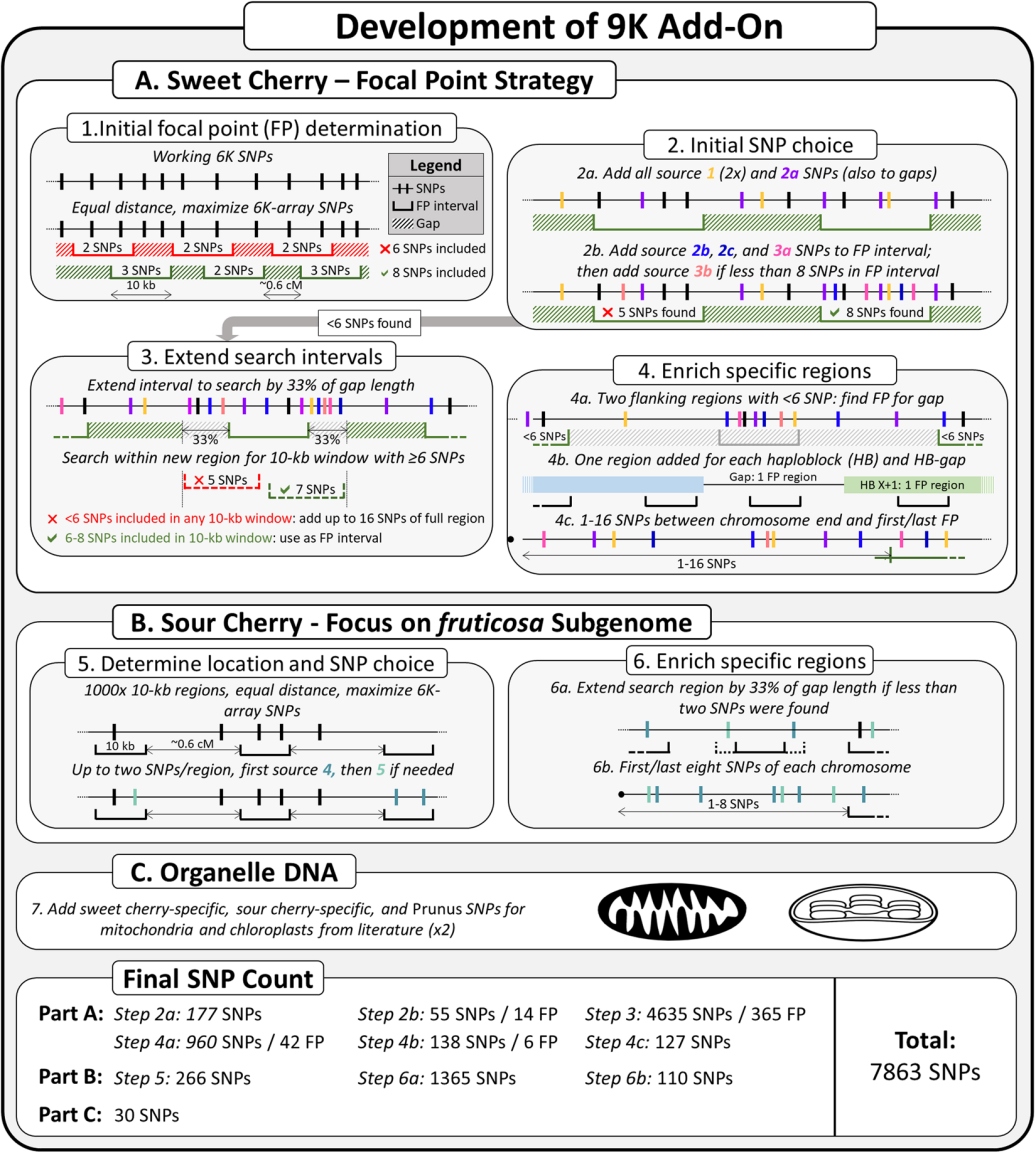
Strategy for the development of the +9K add-on. For sweet cherry, the focal point strategy involved identifying groups of six to eight SNPs within 10-kb windows spaced across the genome. Positions of these 10-kb windows were altered if not enough SNPs were found in the initially proposed windows. Additional SNPs were chosen for regions where no 10-kb windows with 6–8 SNPs could be found. Adequate coverage was also ensured of previously described haploblocks^17^, gaps between haploblocks, and chromosome ends. For sour cherry, focus was put on SNPs representing the *fruticosa* genome and two SNPs were sought in 1000 equidistant 10-kb windows across the genome. Target regions were adjusted where not enough SNPs were found, and adequate coverage of the chromosome ends was ensured. Finally, all reported SNPs representing cherry mitochondria and chloroplasts were included twice.

The final number of sweet cherry SNPs chosen for the +9K add-on was 6942 (Table 1, Supplementary Table S1). All 34 SNPs of source 1 in duplicate and all 172 SNPs from source 2a were incorporated (Fig. 2, Table 2, Supplementary Table S1). Then, 973 initially proposed focal points were identified that maximized the inclusion of reliable polymorphic SNPs from the original SNP array, with 658 original 6K-array SNPs included. Gaps between these initial focal points averaged 0.47 cM. Enough SNPs could be found for only nine of these focal points to obtain 6–8 SNPs each (Table 2, Supplementary Table S1), which provided 55 SNPs to the +9K add-on. After searching additional 10-kb windows for suitability as focal points, another 1709 SNPs across 253 targeted focal points were incorporated into the +9K add-on (Table 2, Supplementary Table S1). An additional 2127 non-focal point SNPs used for the +9K add-on were representing extended search regions for which no focal point with 6–8 SNPs could be found. A further 388 SNPs at 63 targeted focal points and another 1901 non-focal point SNPs for the +9K add-on represented gaps between under-represented regions (Table 2, Supplementary Table S1). Another 74 SNPs at 11 targeted focal points and 163 non-focal point SNPs ensured that all previously characterized U.S. breeding program-based haploblocks and gaps between those haploblocks were represented (Table 2, Supplementary Table S1). For the ends of chromosomes, 77 SNPs in 12 targeted focal points and 196 non-focal point SNPs were included (Table 2, Supplementary Table S1). Finally, 12 non-focal point SNPs were included in the +9K add-on to fill two large physical gaps (Table 2, Table S1). A total of 348 focal points were targeted and consisted of an average of 6.8 SNPs; 2324 SNPs at targeted focal points were part of the add-on while the remaining 52 SNPs were reliable polymorphic SNPs of the original 6K array.

**Table 2:** SNPs chosen and targeted focal points (FP) obtained during each step of development of the +9K add-on. Step 2a to 4d represent SNPs chosen for sweet cherry and are divided into SNPs located in FP and SNPs not located in FP. Steps 5 to 6a represent SNPs chosen for the *fruticosa* genome of sour cherry and no FP were targeted for these SNPs. Step 7 represents SNPs for mitochondrial and chloroplast genomes.

Development step	Number of SNPs			Number of FPs
	In FPs	Not in FPs	Total	
***Sweet cherry***				
Step 2a	—	—	240	—
Step 2b	55	—	55	9
Step 3	1709	2127	3836	253
Step 4a	388	1901	2289	63
Step 4b	74	163	237	11
Step 4c	77	196	273	12
Step 4d	0	12	12	0
***Sour cherry***				
Step 5	—	28	28	—
Step 6a	—	1864	1864	—
Step 6b	—	128	128	—
***Organelle***				
Step 7	—	38	38	—

A total of 2020 SNPs in the +9K add-on targeted the *fruticosa* subgenome of sour cherry (Table 1, Supplementary Table S1). Of the 951 initial 10 kb-sized windows targeted, two SNPs were found for 14 windows, providing 28 SNPs for the +9K add-on (Table 2, Supplementary Table S1). After extending the search windows beyond 10 kb, another 893 windows were found with 2–3 SNPs each, providing another 1821 SNPs to the +9K add-on to target the *fruticosa* subgenome. For each of the remaining 43 windows, only one SNP could be found. A further 128 included SNPs were those targeting chromosome ends (Table 2, Supplementary Table S1).

For organelle DNA, 19 SNPs were obtained from the literature (Table 1). These SNPs consisted of three in mitochondrial DNA and 16 in chloroplast DNA. The inclusion of all organelle SNPs in duplicate brought the total number of SNPs chosen for the development of the add-on to 9000 (Tables 1, 2, Supplementary Table S1).

Development of the +9K add-on added 7863 SNPs (87.3% of targeted SNPs, Table 1) to the original cherry 6K SNP, resulting in the 6+9K cherry SNP array containing 13,559 SNPs. Of the newly added SNPs, 6092, 1741, and 30 SNPs targeted the sweet cherry (and the *avium* subgenome of sour cherry), the *fruticosa* subgenome of sour cherry, and organelle DNA, respectively. For sweet cherry, a total of 2064 SNPs were assigned to targeted focal points, with an average of 6.1 SNPs per focal point (including 6K SNPs). Of the 53 SNPs added in duplicate, 38 occurred twice in the 6+9K array, 12 SNPs were represented a single time, while for three SNPs neither copy made it to the final array.

### Performance of the 6+9K SNP array

For sweet cherry, a total of 2130 SNPs (27%) of the +9K add-on portion of the final 6+9K array were determined to be “reliable polymorphic” (Table 3). The three ASSIsT runs on different subsets of the germplasm resulted in 2249 (29%), 2044 (26%), and 2123 (27%) retained SNPs. Of these SNPs, 1845 (24% of +9K add-on) were reliable polymorphic in all three runs whereas 654 SNPs (8%) were not retained in all runs. After visual inspection of the SNPs that were not always retained, another 283 SNPs (4%) were classified as reliable polymorphic. Of the discarded SNPs, 3398 (43% of the +9K add-on) were reliably clustered but monomorphic and 2335 SNPs (30%) had failed. Most of the retained SNPs for sweet cherry (2093 SNPs, 98% of retained SNPs) were designed for sweet cherry while the remaining 35 retained SNPs were designed to target the *fruticosa* subgenome of sour cherry (Table 3). Of the SNPs targeting sweet cherry, SNPs from source 1 (EST SNPs) were the most successful (90% reliable polymorphic) while intragenic SNPs from both source 2 (sweet cherry GBS SNPs) and source 3 (sweet cherry SNPs identified during the original array’s development) were the least successful (21–23% successful) (Table 3). In addition, mapped SNPs from source 2 were more successful (58% successful) than both unmapped intragenic SNPs of source 2 (41% successful) and intragenic SNPs of source 3 (42% successful).

**Table 3:** Evaluation of +9K add-on SNP performance in sweet cherry and sour cherry. SNPs were classified as either reliable polymorphic, monomorphic, or failed for sweet cherry. For sour cherry, unresolved polymorphic was added as a fourth category. Evaluation of SNP performance is reported for each source and subsource. For each source, the proportion of SNPs within each performance category is given within brackets.

SNP source	Sweet cherry evaluation			Sour cherry evaluation			
	Reliable polymorphic	Monomorphic	Failed	Reliable polymorphic	Unresolved polymorphic	Monomorphic	Failed
***Sweet cherry***	***2093 (34%)***	***2340 (38%)***	***1659 (27%)***	***1091 (18%)***	**3593 (59%)**	***1221 (20%)***	**187 (3%)**
Source 1 (EST)	52 (90%)	2 (3%)	4 (7%)	38 (66%)	15 (26%)	3 (5%)	2 (3%)
Source 2 (GBS)	194 (33%)	293 (50%)	95 (16%)	142 (24%)	146 (25%)	251 (43%)	43 (7%)
*Source 2a (mapped)*	*85 (58%)*	*51 (35%)*	*10 (7%)*	*69 (47%)*	*21 (14%)*	*49 (34%)*	*7 (5%)*
*Source 2b (unmapped intragenic)*	*22 (41%)*	*21 (39%)*	*11 (20%)*	*13 (24%)*	*15 (28%)*	*21 (39%)*	*5 (9%)*
*Source 2c (unmapped intergenic)*	*87 (23%)*	*221 (58%)*	*74 (19%)*	*60 (16%)*	*110 (29%)*	*181 (47%)*	*31 (8%)*
Source 3 (6K array development)	1847 (34%)	2045 (38%)	1560 (29%)	911 (17%)	3432 (63%)	967 (18%)	142 (3%)
*Source 3a (intragenic)*	*1401 (42%)*	*1063 (32%)*	*867 (26%)*	*737 (22%)*	*2126 (64%)*	*406 (12%)*	*62 (2%)*
*Source 3b (intergenic)*	*446 (21%)*	*982 (46%)*	*693 (33%)*	*174 (8%)*	*1306 (62%)*	*561 (26%)*	*80 (4%)*
***Sour cherry***	***35 (2%)***	***1030 (59%)***	***676 (39%)***	***70 (4%)***	**86 (5%)**	***972 (56%)***	**613 (35%)**
Source 4 (*P. fruticosa* GBS)	27 (2%)	974 (58%)	672 (40%)	25 (2%)	70 (4%)	965 (58%)	613 (37%)
Source 5 (6K array development)	8 (12%)	56 (82%)	4 (6%)	45 (66%)	16 (24%)	7 (10%)	0 (0%)
***Organelle***	***2 (7%)***	***28 (93%)***	***0 (0%)***	***0 (0%)***	***0 (0%)***	***30 (100%)***	***0 (0%)***
Mitochondrion	0 (0%)	3 (100%)	0 (0%)	0 (0%)	0 (0%)	3 (100%)	0 (0%)
Chloroplast	2 (7%)	25 (93%)	0 (0%)	0 (0%)	0 (0%)	27 (100%)	0 (0%)
**Total**	**2130 (27%)**	**3398 (43%)**	**2335 (30%)**	**1161 (15%)**	**3679 (47%)**	**2223 (28%)**	**800 (10%)**

According to the peach whole genome sequence (WGS) v2^21^, the average physical distance between reliable polymorphic SNPs in our sweet cherry germplasm was 60 kb and 95% of the gaps between these SNPs were shorter than 250 kb. The largest gaps per chromosome ranged from 917 kb on chromosome 5 to 3488 kb on chromosome 3 (Supplementary Fig. S1). An average of 1.9 SNPs were reliable polymorphic per targeted focal point, with a maximum of eight reliable polymorphic SNPs for some targeted focal points. The target of at least three reliable polymorphic SNPs was obtained for 110 targeted focal points (32%; average of 4.6 SNPs per focal point), 113 targeted focal points had 1–2 SNPs (32%), while for another 125 targeted focal points (36%) no reliable polymorphic SNPs were present. Based on the peach WGS v2, there were additional reliable polymorphic SNPs present, not expected to be within targeted focal points yet with at least three SNPs within 10-kb windows: an additional 224 focal points could be formed by 732 SNPs (an average of 3.5 SNPs per focal point).Thus, the total of focal points with at least three reliable polymorphic SNPs was 237 encompassing 1241 SNPs.

For sour cherry, 1161 SNPs (15%) of the +9K add-on were determined to be reliable polymorphic (Table 3). Most of these reliable polymorphic SNPs targeted the *avium* subgenome: 1091 of the successful SNPs represented the *avium* subgenome while only 70 successful SNPs represented *fruticosa*. Similar to sweet cherry, SNPs from source 1 (EST SNPs) were the most successful (66% reliable polymorphic) while intragenic SNPs from both source 2 (sweet cherry GBS SNPs) and source 3 (sweet cherry SNPs identified during the original array’s development) were the least successful (8–16% reliable polymorphic) (Table 3). For SNPs targeting the *fruticosa* subgenome, those identified during development of the original 6K array were much more successful (66% reliable polymorphic) than SNPs identified with GBS (2% reliable polymorphic).

Only three of the organelle SNPs were polymorphic. Two chloroplast SNPs, pspp_matK_752 and pce_atpB-rbcL_175 (and their duplicates), were able to distinguish between sweet cherry and sour cherry germplasm. A third chloroplast SNP, pav_TPScp1_718 and its duplicate, was polymorphic among sweet cherry individuals but monomorphic for sour cherry individuals. Several further organelle SNPs appeared to include null alleles.

### Updated physical and genetic SNP locations

A unique physical location in the cherry WGS was detected for each of the 7366 SNPs (54%) of the 6+9K SNP array: 4562 SNPs (80%) of the original 6K SNP array and 2804 SNPs (36%) of the +9K add-on had a unique location (Supplementary Table S2). For the remaining SNPs, an average of 6.7 locations, with a maximum of 738, were identified for sweet cherry SNPs while an average of 5.1 locations with a maximum of 777 were identified for *fruticosa* subgenome SNPs (Supplementary Table S2). An average of 1.3 locations in the cherry WGS were identified for each reliable polymorphic SNP in sweet cherry, with a maximum of 53 locations. No physical location could be found for 308 reliable polymorphic SNPs of sweet cherry (8%) and another 279 of these SNPs (7%) were uniquely anchored to chromosome zero and thus were not assigned to one of the 8 pseudomolecules. In contrast, an average of 7.2 and 2.2 locations in the cherry WGS were identified for monomorphic and discarded SNPs in sweet cherry, respectively. No location could be found for 495 (12%) and 845 (16%) of the monomorphic and failed SNPs, respectively. The average physical distance between polymorphic reliable SNPs of sweet cherry was 63.5 kb and 95% of such gaps were shorter than 250 kb. The largest gap per chromosome ranged from 575 kb on chromosome 6 to 1472 kb on chromosome 3. Based on the cherry WGS^22^, there were 235 focal points encompassing 890 SNPs (3.8 reliable polymorphic SNPs per focal point).

**Figure 3:**
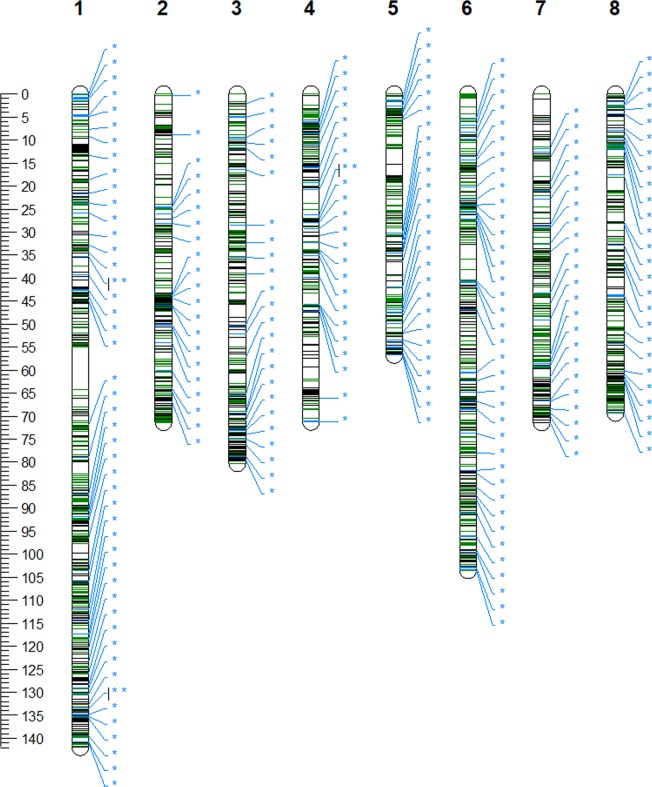
Estimated genetic coverage of array SNPs. SNPs from the original array are shown in black. SNPs not belonging to a focal point are shown in green, while SNPs within 10-kb windows according to the cherry whole genome sequence^22^ are shown in blue. Positions of focal points are marked with blue asterisks.

Based on estimated genetic positions, the average genetic distance between reliable polymorphic SNPs in sweet cherry was 0.18 cM (Fig. 3, Table S3). The longest gap was 9.26 cM, on chromosome 1, while the second longest gap was much smaller: 3.56 cM on chromosome 7. Only 3% of the gaps (121 gaps) were larger than 1 cM (Table S3). Based on the cherry WGS, the average distance between two focal points was 2.8 cM and the largest distance was 26.99 cM on chromosome 1. A total of 1620 and 458 reliable polymorphic SNPs were added to 172 of 196 (88%) previously determined haploblocks and 139 of 187 (74%) gaps between those haploblocks (Table S3). A total of 50 reliable polymorphic SNPs were positioned at the proximal ends of chromosomes 1, 2, 3, 4, 6, and 7 and at the distal ends of chromosomes 2, 3, 4, 6, and 8, with at least one reliable polymorphic SNP per chromosomal extremity.

### Application to a fruit firmness QTL

**Figure 4:**
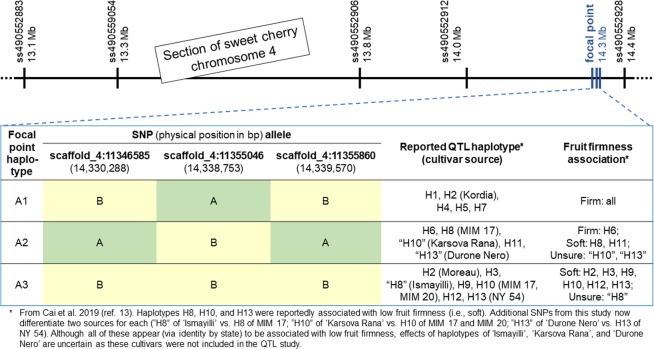
SNP location according to the cherry whole genome sequence^22^, SNP haplotypes, and associated QTL haplotypes of a focal point within a fruit firmness QTL (*qP-FF4.1*) on chromosome 4 previously characterized with five SNPs^12^. The focal point with three SNPs was positioned 63 kb upstream from SNP ss49055298 (the SNP most associated with fruit firmness^12^).

A three-SNP focal point was located 63 kb upstream of SNP ss490552928, the SNP most associated with the targeted fruit firmness QTL^12^ (Fig. 4). Of the eight possible allelic combinations for this focal point, only three were observed in the sweet cherry germplasm used: “BAB” (A1), “ABA” (A2), and “BBB” (A3). The first focal-point haplotype, A1, was linked in coupling phase to the firm-associated QTL haplotypes of H1, H2 originating from ‘Kordia’, H4, H5, and H7 as defined by Cai *et al*.^12^. A2 was in coupling phase with the firm-associated QTL haplotype H6 and soft-associated QTL haplotypes H8, H10, H11, and H13. A3 was in coupling phase with the soft-associated QTL haplotypes H2 originating from ‘Moreau’, H3, H8, H9, H10, H12, and H13. Thus, A1 was exclusively linked to firm-associated QTL haplotypes while A2 and A3 were almost exclusively linked to “soft” QTL haplotypes with H6 being the only “firm” QTL haplotype linked in coupling phase to A2. In addition, one SNP of the focal point, scaffold_4:11355046, was also almost completely predictive because the “A” allele of this SNP was linked in coupling phase to the A1 haplotype of the focal point (and thus to the “firm” QTL haplotypes) and the “B” allele of this SNP was linked in coupling phase to the A2 and A3 haplotypes of the focal point (and thus to “soft” QTL haplotypes with the exception of H6).

## Discussion

A +9K add-on developed for the cherry 6K SNP array more than doubled the available number of reliable, polymorphic SNPs for sweet cherry and increased the number of reliable, polymorphic SNPs for sour cherry by more than a thousand. The use of already-available data to choose prospective array SNPs bypassed the need to obtain new sequence data and perform *de novo* SNP discovery. Choosing to add additional SNPs to the original SNP array rather than developing a new array provided an effective way to improve on the utility while maintaining the strengths of the original cherry 6K SNP^2^. The approach also ensured that a common genotyping tool is available for the cherry research community, supporting collaboration. Other genotyping techniques, such as genotyping by targeted sequencing (e.g., Allegro Targeted Genotyping^23^) exist and might have been cheaper per data point but overall cost per sample would still be higher. In addition, minimum sample requirements for these techniques are generally much higher than those for the SNP array, and not feasible in the context of cherry breeding programs. Finally, these techniques would also have had to be validated for cherry whereas Illumina’s beadchip technology was already confirmed to work for cherry. Overall, development of the +9K add-on provided a cost-effective way to improve the original array. For example, in sweet cherry most individuals genotyped are expected to have data for 580–890 additional reliable polymorphic SNPs attributed to the +9K add-on. Because the resulting new array contains all SNPs from the original cherry 6K SNP array supplemented by the +9K add-on, it is called the 6+9K cherry SNP array.

The focal point strategy for add-on array design was effective. Initially proposed focal points were able to be evenly spread throughout the genome and maximized the use of reliable polymorphic SNPs already present on the original array. Many of the initially targeted windows did indeed contain at least one reliable, polymorphic SNP from the original SNP array. However, the targeted SNP number per focal point could not be achieved for most windows. A reason could be the relatively low availability of SNPs to choose from. Although multiple sources of sequence data and SNP discovery were used^2,19,20,24^, an average of only 2.7 SNPs and 0.6 SNPs were available per 10-kb window in the initial and final SNP pools, respectively. In contrast, more than one million SNPs were reported in the cherry WGS^22^, equivalent to an average of ~29 SNPs per 10-kb window. It is not clear how many of these SNPs would be located within 20 bp of each other, which was one of the criteria we used in filtering to improve genotyping success rate. Thus, although a limited number of SNPs were available for this study (Supplementary File S1, part 1), enough SNPs might be present in the cherry genome for a focal point strategy across the genome.

The higher proportion of failed SNPs in +9K add-on compared to what was reported for the original 6K SNP array by Peace *et al*. (27% vs. 5% respectively)^2^ was mostly associated with the *fruticosa*-targeted sour cherry SNPs. Specifically for sweet cherry, the proportion of SNPs performing as reliable and polymorphic in the add-on was similar to that observed for the original SNP array^2^. In sour cherry, the success rate of SNPs targeting the *fruticosa* subgenome (4%) was much lower than reported for the original array (43%)^2^, which was mostly because of the very low success rate of SNPs coming from GBS data of *P. fruticosa* individuals^20^ and the high proportion of these SNPs used here to target the *fruticosa* subgenome. SNPs in the add-on that were identified during the development of the original array had a higher failure rate than the SNPs of the original array although these SNPs were discovered together. Thus, aside from SNP source, other reasons must exist for this observed difference in failure rate, such as differences in evaluation of SNP performance from genotyping. Indeed, SNPs were inspected visually for the 6K array^2^, while ASSIsT^25^ was used for the +9K add-on. The use of different sweet cherry germplasm for the evaluation might also explain the different failure rates (Supplementary File S1, part 2). Nevertheless, when ASSIsT was used on the germplasm of this study, the failure rate for the SNPs of the original cherry 6K SNP array (10%, results not shown) remains lower than for the SNPs of the +9K add-on and thus some unknown factor increased the failure rate for the add-on.

Differences in success rate were observed among experimental sources of *in silico* SNPs. Both sources of previously mapped SNPs (source 1^24^ and source 2a^19^, Fig. 1) had the highest success rates (47.3–89.7%), both in sweet and sour cherry. Similarly, previously validated SNPs had high success rates between (92%-96%) during the validation of the cherry 6K^2^, apple 20K^16^, and pear 70K^26^ SNP arrays. Thus, SNPs validated in previous studies have provided the most effective targets during the development of SNP arrays. Furthermore, the higher success rate observed for intragenic SNPs compared to intergenic SNPs in both sweet cherry and both subgenomes of sour cherry is consistent with results from the original cherry 6K^2^, the apple 8K^14^ and 480K^27^, and peach 9K^15^ SNP arrays. This indicates that the strategy to target intragenic SNPs used for the original cherry 6K SNP array was appropriate^2^. For sweet cherry, similar rates of reliable polymorphic SNPs between SNPs identified through whole genome re-sequencing and the GBS bioinformatics pipeline indicate that both are effective strategies to identify polymorphic SNPs. However, cherry SNPs identified through GBS were more often monomorphic and failed less. Similarly, GBS SNPs on the apple 480K SNP array were more often monomorphic^27^. Higher monomorphic rates for GBS SNPs could indicate that this method is more prone to false positives during SNP discovery. The reduced failure rate could be explained as follows: although overall coverage by GBS might have been lower^19^, regions targeted by GBS might have a higher local coverage than that achieved by low-coverage whole-genome genome re-sequencing^2^. As such, closely located SNPs (i.e., targeted SNPs with other SNPs in the probe-binding region) might have been more readily detected for GBS sources, and in the subsequent filter steps these closely positioned SNPs would have been removed. Thus, it would be less likely for SNPs identified through GBS to have SNPs in the probe-binding region, reducing their failure rate. For sour cherry, our strategy of using GBS data of *P. fruticosa* individuals as a proxy for the *fruticosa* subgenome of sour cherry was unsuccessful. Several possible reasons exist for the failure of these SNPs (Supplementary File S1, part 3) but most SNPs probably failed because of differences between the *P. fruticosa* germplasm sequenced in GBS and the *P. fruticosa* germplasm that is ancestral to sour cherry. Because of this low success rate, the desired outcome of more equal representation of each subgenome of sour cherry was not achieved. Instead, the *avium* subgenome of sour cherry has become even more overrepresented compared to the *fruticosa* subgenome. Finally, the success rate of organelle SNPs was low and most of them were monomorphic. Previously found SNPs might not segregate in our population, might have been false positives, or their flanking sequence might have been determined incorrectly.

The availability of the sweet cherry WGS^22^ enabled determination of the physical location of all SNPs on the 6+9K SNP array. However, almost half of the SNPs could not be uniquely mapped to this cherry WGS. Thus, although SNPs were originally filtered to uniquely map to the peach WGS^21^, this did not ensure that they also mapped uniquely to the cherry WGS^22^. However, most of the multi-location SNPs were classified as either failed or monomorphic and are less likely to be important to users of the 6+9K SNP array. On the other hand, most reliable polymorphic SNPs, which are more likely to be important to users, did map uniquely. In addition, some inconsistencies remain between physical and genetic positions. Because genetic positions for SNPs were determined earlier based on the peach WGS, the observed inconsistencies between genetic and physical position might be explained by non-syntenic regions between the peach and cherry genomes. However, an extensive workflow has been used to resolve any map issues for SNPs from the original cherry 6 K SNP array using U.S. representing sweet cherry germplasm^17^, leading to high confidence in genetic positions of the SNPs, even if they were originally based on the peach WGS. Furthermore, mapping populations have been used to determine genetic positions of SNPs on the cherry 6K SNP array^5^. These maps did not rely on the peach WGS but still about 5% of the SNPs map to a different chromosome than indicated by the sweet cherry WGS. The combined observations of many reliable polymorphic SNPs having multiple physical locations in the reported cherry WGS^22^ and inconsistences between this WGS and validated genetic maps suggests some issues in the assembly of the current sweet cherry WGS. The germplasm used in this study will be put through the same extensive workflow for data curation to result in accurate genetic locations for the SNPs of the +9K add-on. From there, the 6+9K array could help improve the assembly of the sweet cherry WGS.

The +9K add-on increased marker density compared to the original 6K array, although some gaps remain. Indeed, 2132 and 1161 new polymorphic SNPs were identified for sweet cherry and sour cherry, respectively. The number of polymorphic SNPs can change depending on the germplasm used: for example, roughly 13% of SNPs retained by ASSIsT^25^ in each run were not retained when using a different subset of the sweet cherry germplasm. Similarly, some SNPs from the original 6K SNP array retained for this germplasm were discarded in previously used germplasm^17^. Thus, some germplasm-dependent performance is expected and, although the performance of 6+9K SNPs in this study can be used as a guideline, we recommend that SNP performance is checked again in one’s own germplasm using ASSIsT^25^ as part of a more elaborate curation workflow^17^. Each chromosome for sweet cherry has at least one physical gap larger than 500 kb and, based on the peach WGS v2, the largest gap on most chromosomes was even larger than 1 Mb. However, these large gaps were found to be in or very close to the centromeric regions of the peach WGS^21^ (Supplementary Fig. S1) and probably the cherry genome although sweet cherry centromeric positions have not yet been reported. These large physical gaps therefore correspond to small genetic gaps and thus higher SNP density in these large physical gaps are unlikely to increase precision in mapping QTLs and identification of candidate genes^28^. Based on genetic positions, most gaps were smaller than the target of 1 cM maximum. A single large gap of 9.3 cM was observed that corresponds to an estimated 140.7 kb in the peach WGS v2^21^ or 102.7 kb in the cherry WGS. Given the small physical gap size, the large genetic gap is likely due to an error in the scaffold map and will most likely not negatively influence locating QTLs for agronomic traits in cherry. Frequency of recombination in the genotyped germplasm could help deduce the true size of this gap. Because a diverse germplasm was used in this study, more and larger gaps might be present in reported biparental mapping families. Nevertheless, gaps in the genetic map are estimated to remain relatively small for biparental families. Indeed, for any given parent genotyped with the 6+9K array, the average gap size between reliable polymorphic SNPs is estimated to range between 0.39 cM and 0.73 cM and 95% of gaps are estimated to be less than 3.4 cM (results not shown). Furthermore, the largest gap size is estimated to range between 11.4 cM and 25.1 cM, except for in some highly homozygous individuals. In contrast, the average distance between markers in existing high-density cherry maps ranges between 0.9 cM and 3.0 cM, 5% of the gaps are larger than 6.1 cM and the largest gap ranges between 10.9 cM and 50.7 cM^4,5^. Thus, the +9K add-on is expected to improve available maps for cherry.

The improved cherry 6+9K SNP array can provide additional information to breeders, geneticists, and allied scientists as exemplified by focal-point characterization of a fruit firmness QTL^12^. The three-SNP focal point helped differentiate between identical QTL haplotypes from different germplasm sources. For example, the H2 haplotype of ‘Kordia’ and its descendants was linked in coupling phase to the “BAB” focal point haplotype while H2 of ‘Moreau’ and its descendants was linked in coupling phase to the “BBB” focal point haplotype. Interestingly, these two H2 haplotypes seem to be associated with differing effects on firmness (Supplementary Table S2 of Cai *et al*.^12^) but this difference in effect was not reported in the publication itself^12^, probably because no distinction between the two H2 haplotypes was possible. In addition, extended haplotypes across this region^17^ also differed between ‘Kordia’ and ‘Moreau’, providing more evidence that these two QTL alleles could indeed be associated with different QTL effects. Because of the availability of additional SNPs within the QTL interval, these two H2 haplotypes can now be distinguished, ensuring that the correct effect will be assigned to each haplotype and that breeders have more confidence when making decisions using these haplotypes. The focal point haplotypes also seem to be almost completely predictive for the fruit firmness alleles of the QTL. The most highly associated SNP and the three SNPs of the focal point are therefore excellent targets for use in DNA-informed breeding except for H6-containing germplasm. The presence of an almost-predictive focal point also warrants further analyses to determine why H6 is the exception to further increase knowledge of this important locus.

SNP array users can group SNPs into multi-allelic markers based on the provided focal points or other criteria. Although focal point assignment should be fixed within a single study, SNP assignment to focal points depends on the genome used and users can adjust focal point assignment in future studies as improved genome versions are released. Furthermore, users can determine their own focal-point window size based on physical or genetic positions (e.g., 15 kb or 1 cM, respectively). For example, in peach, informative SNPs of the 9K SNP array have been grouped in 1 cM windows^29^. Rather than grouping SNPs based on a fixed window size, they can be grouped into haplotypes based on linkage disequilibrium or historic recombination events^28^. For example, haplotypes based on historic recombination events have been determined for U.S. breeding germplasm of apple, peach, and sweet cherry using the apple 8K, peach 9K, and cherry 6K SNP arrays, respectively^17^. The new, improved genetic map provided by this study enables improved precision of determining genomic positions of recombination events, in turn improving haploblock informativeness for sweet cherry germplasm and fine-mapping of QTLs and candidate genes. The additional SNPs within each haploblock should better distinguish among haplotypes from different ancestral sources or increase confidence that haplotypes are indeed the same with a common ancestral source. As such, the 6+9K array will greatly help with pedigree reconstruction of the world’s sweet cherry ger-mplasm and genetic dissection of valuable traits.

## Conclusion

A new, improved genome-wide SNP array is now available for sweet and sour cherry. This 6+9K SNP array includes all SNPs of the original cherry 6K array supplemented by an additional 7863 SNPs. Previously validated, mapped SNPs performed the most reliably followed by non-validated intragenic SNPs. The add-on mostly contributes additional information for sweet cherry and the *avium* subgenome of sour cherry, although additional information is also provided for the *fruticosa* subgenome of sour cherry and for some chloroplast polymorphism. The use of previously identified SNPs was a cost-effective way to improve the original array. The improved array will enable more precise genetic analyses and help improve our genetic understanding of worldwide cherry germplasm.

## Materials and Methods

### SNP sources

Five sources of SNPs were used: three for sweet cherry and two for sour cherry (Fig. 1). For sweet cherry, the initial pool of SNPs consisted of 1) SNPs from EST data^24^, 2) SNPs identified *in silico* through GBS used to construct sweet cherry linkage maps^19^, and 3) SNPs identified *in silico* from re-sequencing 16 sweet cherry individuals during development of the original cherry 6K SNP array (Fig. 1). For sour cherry, only SNPs targeting the *fruticosa* subgenome were considered because sweet cherry SNPs were concluded to sufficiently represent the *avium* subgenome of sour cherry^2^. The initial pool for sour cherry consisted of 4) SNPs identified *in silico* through GBS of 32 *P*. *fruticosa* individuals from three geographically separated natural populations in the Vojvodina Province, Republic of Serbia^20^ (Fig. 1) and 5) *fruticosa* subgenome-specific “Stage 1” filtered SNPs identified *in silico* during development of the original cherry 6K SNP array^2^. The *P*. *fruticosa* SNPs obtained through GBS^20^ were used because they were considered to represent polymorphism in the *fruticosa* subgenome of sour cherry in the same way that SNPs of sweet cherry were successfully determined to represent the *avium* subgenome^2^.

Final groups were then created to choose SNPs for the +9K add-on. First, the position of SNPs according to the peach WGS v2^21^ was determined using BLAST+ ^30^ as no cherry WGS was available at the time. Then, sweet and sour cherry SNPs were removed from their initial pools if any other sweet or sour SNP, respectively, was detected within 20 bp according to the peach WGS v2^21^ (Fig. 1). Next, ‘Stage 2 filtering’ was applied as described by Peace *et al*.^2^ except that both intragenic and intergenic SNPs were kept for sweet cherry. For sour cherry, only intragenic SNPs were kept as they had been incorrectly removed for the *fruticosa* subgenome during the development of the original cherry 6K SNP array. In addition, SNPs already on the 6K SNP array were removed. The obtained pool of SNPs was then divided into five groups according to their initial source: 1) sweet cherry SNPs from ESTs^24^, 2) sweet cherry SNPs identified *in silico* through GBS^19^, 3) sweet cherry SNPs identified *in silico* during the original 6K array’s development^2^, 4) *P. fruticosa* SNPs for sour cherry identified *in silico* through GBS^20^, and 5) *fruticosa*-subgenome specific SNPs for sour cherry identified *in silico* during the original 6K array’s development^2^. SNPs of sources 2 and 3 were further divided into the following five subgroups: 2a) mapped GBS SNPs^19^, 2b) unmapped intragenic GBS SNPs^19^, 2c) unmapped intergenic GBS SNPs^19^, 3a) intragenic SNPs from the original 6K array’s development^2^, and 3b) intergenic SNPs from the original 6K array’s development^2^. These groups of suitable SNPs were supplemented with previously reported organelle SNPs: 16 chloroplast SNPs^31,32^ and 3 mitochondrial SNPs^31^. Five, six, and two chloroplast SNPs had been identified in sweet cherry, sour cherry and both, respectively^31,32^. The remaining three chloroplast SNPs differentiated between cherry species. SNP names used those of their original publications as explained in Supplementary File S2.

### Sweet cherry SNP choice

The strategy to choose SNPs for sweet cherry is described below and in Fig. 2. A focal point strategy was used that sought to assign groups of 6–8 SNPs to 10-kb windows spaced throughout the genome. In the first step, positions of proposed focal points were determined throughout the genome so that adjacent focal points were separated by 1 cM or less^17^ (to avoid large genetic gaps) and so that as many reliable polymorphic SNPs as possible from the original SNP array were located within the focal-point windows. The genetic positions of the focal points were then converted to physical positions in the peach WGS v2^21^ as no cherry genome was available at the time. In the second step, two copies of SNPs from source 1 (EST SNPs) and a single copy of SNPs from source 2a (mapped GBS SNPs) were included in the +9K add-on regardless whether they were at a focal point, because of their considered high information value for cherry genetics research. Then, a total of three reliable polymorphic SNPs per focal point was targeted to be on the final SNP array. To ensure this target was achieved, 6–8 SNPs were targeted during the development of the +9K add-on, including reliable polymorphic SNPs from the original SNP array and already chosen SNPs from sources 1 and 2a. To reach eight SNPs, priority was given to SNPs from source 2b (unmapped intragenic GBS SNPs), 2c (unmapped intergenic GBS SNPs), and source 3a (intragenic SNPs from the original array’s development). Where less than six SNPs were found for a focal point, SNPs from source 3b (intragenic SNPs from the original array’s development) were chosen to achieve 6–8 SNPs. In the third step, each remaining proposed focal point with less than six SNPs was replaced by a new one targeting a nearby 10-kb window and containing 6–8 SNPs according to the criteria of the first two steps. Only a limited interval was searched: search interval on each side of the original focal point consisted of a third of the physical distance between the focal point’s original location and the location of the adjacent focal point (according to peach WGS v2^21^). If no 10-kb window with 6–8 SNPs was found, a diffuse focal point was created with up to 16 SNPs in the total search interval (> 10 kb) were chosen to represent that genomic region. In the fourth step, specific genomic regions were targeted to improve their coverage. Where two adjacent search intervals did not yield a focal point, the gap between the two regions were searched for a 10-kb focal point with 6–8 SNPs. If no suitable focal point was found in this gap, up to 16 SNPs diffusely spanning the region were chosen. Secondly, we ensured that the +9K add-on included SNPs within each of the previously defined^17^ haploblocks and gaps between them, where such haploblocks are intervals of no known historical recombination within the pedigrees of U.S. selected cherry breeding material. Finally, up to 16 SNPs were chosen between the ends of each chromosome and the first or last focal point of that chromosome, respectively.

### Sour cherry SNP choice

Similarly to sweet cherry, the sour cherry genome was divided into 1000 intervals (~0.6 cM each) and a 10-kb window was determined within each interval that maximized the number of reliable polymorphic SNPs from the original SNP array for the *fruticosa* subgenome within these 10-kb windows according to peach WGS v2^21^ (Fig. 2). Each 10-kb window was filled with up to two SNPs (including the original array’s reliable polymorphic SNPs) using SNPs from source 4 (*P. fruticosa* GBS SNPs). Any 10-kb window not containing two SNPs was further filled with SNPs from source 5 (SNPs identified during the original array’s development). For each remaining 10-kb window with less than two SNPs, the search interval was extended on each side by a third of the distance between itself and the adjacent 10-kb windows. This extended window was then filled with up to two SNPs, again giving priority to the SNPs from source 4. Finally, the eight available SNPs positioned at the ends of each chromosome were included.

### Organelle SNP choice

Reported polymorphic organelle SNPs were included for their use in pedigree reconstruction (Fig. 2): given that organelles are only passed on by the mother to their offspring, these SNPs should help determine whether an individual’s newly found parent is the mother or the father. All organelle SNPs were added twice due to their low number and importance in pedigree reconstruction, given the expectation that a small proportion of submitted SNPs is lost during manufacture of the final SNP array by Illumina.

### **Evaluation of the** +**9K add-on**

#### Tissue collection, DNA extraction, and iSCAN

For sweet cherry, leaf tissue of 502 individuals was collected. The germplasm consisted of 79 individuals (cultivars and unselected seedlings) already genotyped with the cherry 6K SNP array^2^ as a subset of a larger set of individuals representing U.S. cherry breeding germplasm^33^, 81 parents of the Pacific Northwest Sweet Cherry Breeding Program (PNWSCBP) at Washington State University, 41 selections and 146 unselected seedlings of the PNWSCBP, and 38 accessions from the National Clonal Germplasm Repository in Davis, CA, USA. For sour cherry, leaf tissue of 380 individuals was collected. This group consisted of 15 cultivars, 7 selections, and 358 unselected seedlings from Michigan State University’s sour cherry breeding program.

Genomic DNA of 384 sweet cherry individuals was extracted by the Clemson University Genomics Institute (Clemson University, Clemson, SC, USA) using a DNA extraction buffer containing 100 mM Tris pH 8.0, 50 mM EDTA pH 8.0, 500 mM NaCl, 1% SDS, 1% PVP40, 500 μg/ml proteinase K, 0.13% diethyldithiocarbamate, 0.1% ascorbic acid, and 0.1 DDT. Homogenized tissue was incubated in the extraction buffer at 65 °C for 30 minutes, then cooled to −20 °C for 15 minutes. Subsequently, a half-volume of 4 °C 6 M ammonium acetate was added, samples were stored at −20 °C for 15 minutes and then centrifuged at 4,200 rpm for 20 minutes. A 3:5 volume of isopropanol and 10 mg/ml glycogen were added to the filtered supernatant and DNA was allowed to precipitate for at least 30 minutes before a 30-minute centrifugation at 4000 rpm. The supernatant was discarded and the pellet washed with 4 °C 70% ethanol before resuspension in 50 µl of DNase-free water. Finally, 1 µl of 1 mg/ml RNase was added. Genomic DNA of the remaining 118 sweet cherry individuals and all sour cherry individuals was extracted using the E-Z 96 Tissue DNA Kit (Omega Bio-Tek, Inc., Norcross, GA, USA). DNA was quantitated with the Quant-iT PicoGreen Assay (Invitrogen, Carlsbad, CA, USA) and DNA concentrations were adjusted to a minimum of 50 ng/µl, in 5 µl aliquots. DNA samples were run on the cherry 6K SNP array with +9K add-on (cherry 6+9K SNP array) with an iSCAN at the Research Technology Support Facility of Michigan State University (East Lansing, MI, USA), following the manufacturer’s protocol (Illumina Inc.).

#### Genotypic data obtainment and determination of reliable SNPs

For sweet cherry, initial genotypic data were obtained through the genotyping module of Illumina’s GenomeStudio software. The quality of data both for each individual and each SNP was then evaluated using steps 1a and 1b, respectively, of the curation workflow described by Vanderzande *et al*.^17^. Briefly, low-quality and non-diploid samples were identified using a sample’s B-allele frequency^17,34^ and discarded, and ASSIsT^25^ was used to assess the quality of genotype clustering, with discarding of monomorphic and failed SNPs^17^. iSCAN data were received in two batches which provided an opportunity to check the extent to which SNP scoring quality was batch-dependent. ASSIsT was used on the two subsets of the genotypic data as well as on all genotypic data at once and results compared as follows. SNPs that passed all ASSIsT runs were classified as “reliable polymorphic”. SNPs that passed at least one but not all ASSIsT runs were visually evaluated for their clustering quality and manually classified as “reliable polymorphic”, “monomorphic”, or “failed”.

For sour cherry, initial genotypic data were obtained through the polyploid clustering module of GenomeStudio. Because sour cherry individuals are tetraploid, and thus up to five genotype clusters were expected for a SNP, ASSIsT was not used to evaluate SNP performance. Instead, SNP performance was manually checked in GenomeStudio by visually examining SNP cluster plots. A fourth classification, “unresolved polymorphic”, was used for polymorphic SNPs that exhibited ambiguous clusters^2^.

### SNP physical positions in the cherry genome

After establishment of the +9K add-on SNPs, a reference sweet cherry whole genome sequence became available^22^. SNP physical positions in the sweet cherry WGS were determined for all SNPs on the 6+9K SNP array using BLAST+ ^30^. Only BLAST hits that covered at least 80% of a SNP’s flanking sequence and with at least a 90% identity between flanking sequence and the sweet cherry WGS were retained. When a SNP was indicated to have more than one possible location in the cherry WGS, flanking SNPs were considered and the BLAST hit that best preserved synteny between the cherry and peach WGS v2 was chosen as the SNP’s position in the cherry WGS. When no BLAST hits preserved synteny, the BLAST hit with the highest score was chosen as the SNP’s position in the cherry WGS. Finally, the number of BLAST hits were correlated with SNP performance (reliable polymorphic vs. monomorphic vs failed).

### SNP genetic positions in the cherry genome

Genetic positions were estimated for reliable polymorphic SNPs in sweet cherry to create a new genetic map. The genetic map consisting of reliable polymorphic SNPs from the original cherry 6K SNP array^17^ was used as a scaffold. Genetic positions of SNPs not in the scaffold map were estimated such that new SNPs had the same position in the genetic map relative to flanking SNPs as in the physical genome. In case of inconsistencies between physical and genetic order (e.g., inversions of chromosomal segments and different chromosome assignments), SNPs without genetic positions were considered as consistent with the closest SNP (based on physical distance) in the genetic map.

### Application to a fruit firmness QTL

To illustrate the advantage of additional SNPs on the array and a focal point strategy, the genomic region of an important fruit firmness QTL on chromosome 4^12^ was searched for available focal points and the focal point closest to the SNP reported to be most significantly associated with fruit firmness^12^ was chosen. Genotypic data for the SNPs belonging to the specified focal point as well as the five SNPs identified by Cai *et al*.^12^ were extracted from the data set and curated for errors using the workflow described by Vanderzande and co-authors^17^. The SNP data of the focal point and five SNPs identified by Cai *et al*.^12^ were phased by FlexQTL^35^ to determine haplotypes for the focal point (labeled A1-A3) and to determine coupling-phase linkages between previously identified haplotypes for the QTL^12^ and the focal point haplotypes.

## Supplementary information

Supplementary Figure S1.

Supplementary Information 1.

Supplementary Information 2

Supplementary Dataset 1.

Supplementary Dataset 2.

Supplementary Table S1.

Supplementary Table S2.

Supplementary Table S3.

## Data Availability

SNPs previously identified and chosen for the +9K add-on were made available through their respective publications. The 6+9 KSNP array and its manifest files are available from Illumina Inc and manifest files are also available in Supplementary Files S3-S4. Flanking sequences of each SNP of the +9K add-on is available in Supplementary Table S1. All final SNP information is available through the Genome Database for Rosaceae (https://www.rosaceae.org/publication_datasets).
