# Supplementary material for: The cherry 6+9K SNP array: a cost-effective improvement to the cherry 6K SNP array for genetic studies
Source: Sci Rep. 2020 May 6;10:7613. doi: 10.1038/s41598-020-64438-x (PMC7203174; doi:10.1038/s41598-020-64438-x)

Title: The cherry 6+9K SNP array: a cost-effective improvement to the cherry 6K SNP array for genetic studies

Authors: Stijn Vanderzande, Ping Zheng, Lichun Cai, Barac Goran, Ksenija Gasic, Dorrie Main, Amy Iezzoni, Cameron Peace

Supplementary Figure S1: Physical size of the gaps between reliable polymorphic markers for each chromosome according to the physical location of the gap on the chromosome. Orange arrows indicate the approximate centromeric positions according to the peach genome v2[21].

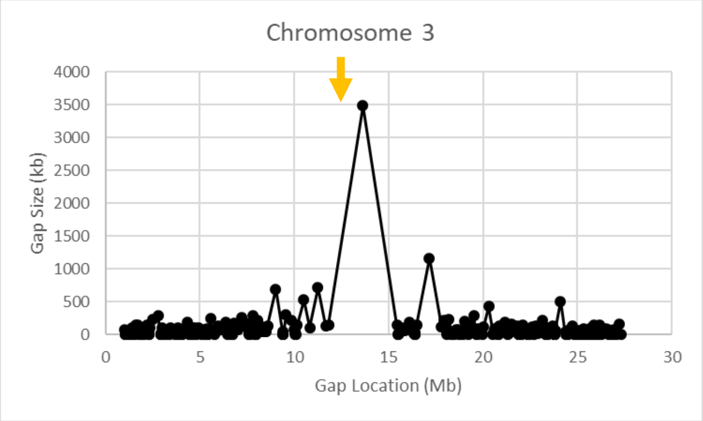

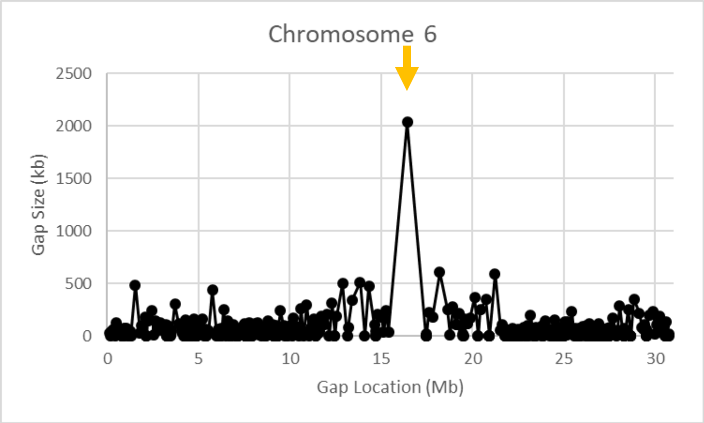

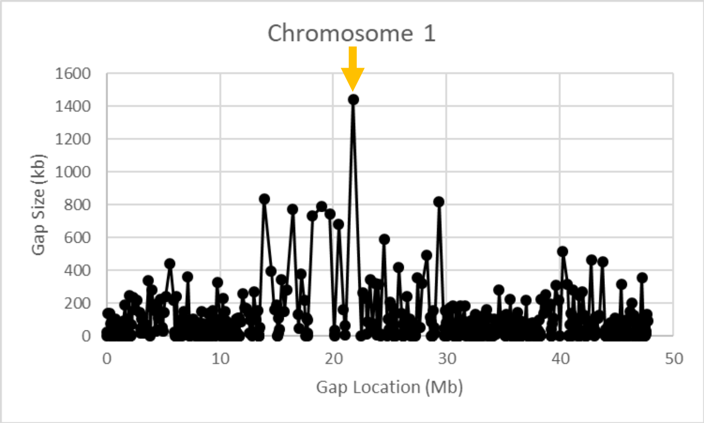

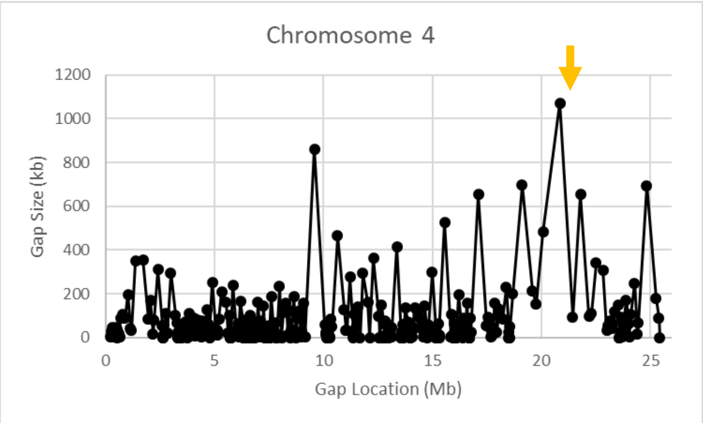

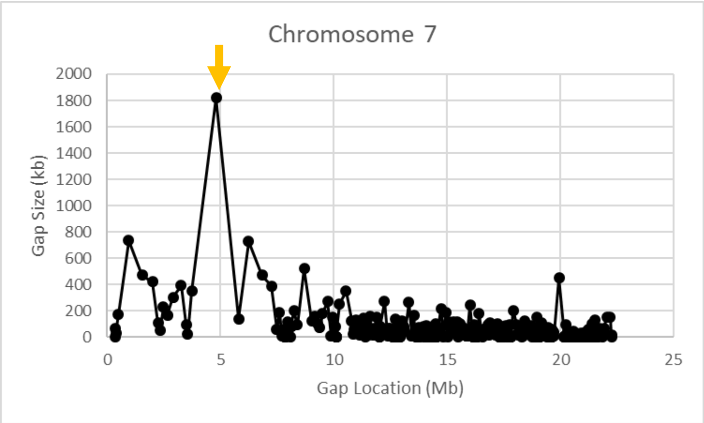

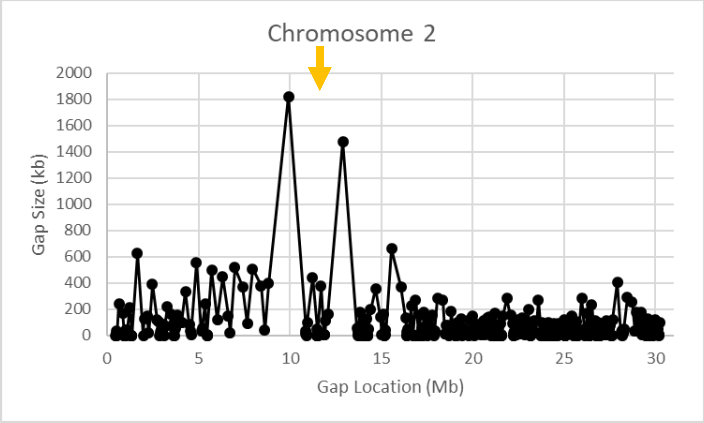

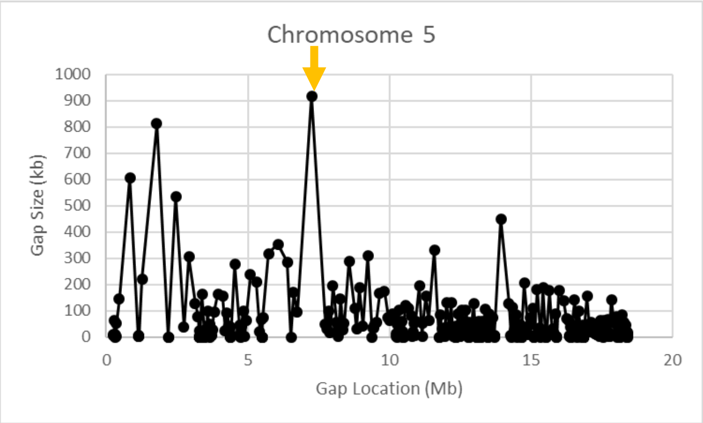

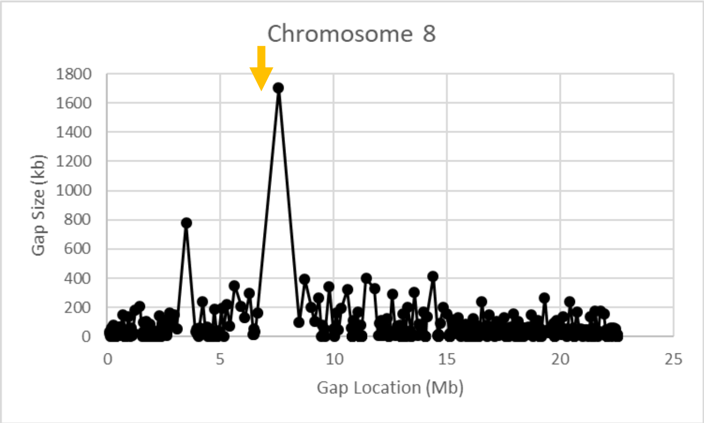

Supplement: Supplementary file 1 — Supplementary Figure S1. [file 41598_2020_64438_MOESM1_ESM.pdf]
