# Supplementary material for: The cherry 6+9K SNP array: a cost-effective improvement to the cherry 6K SNP array for genetic studies
Source: Sci Rep. 2020 May 6;10:7613. doi: 10.1038/s41598-020-64438-x (PMC7203174; doi:10.1038/s41598-020-64438-x)
Supplement: Supplementary file 2 — Supplementary Information 1. [file 41598_2020_64438_MOESM2_ESM.pdf]

The cherry 6+9K SNP array: a cost-effective improvement to the cherry 6K SNP array for genetic studies

Stijn Vanderzande, Ping Zheng, Lichun Cai, Barac Goran, Ksenija Gasic, Dorrie Main, Amy Iezzoni, Cameron Peace

Supplementary File S1: Further elaboration of some discussion points regarding SNP performance

1. Possible reasons for the low number of SNPs available in this study

The limited availability of SNPs for this study might have been caused by the low coverage obtained by sequencing efforts or because reads were mapped against the peach whole genome sequence (WGS)[1,2]. In apple, an average coverage of 43× was obtained for the development of the apple 20K SNP array which also used a focal point strategy. In contrast, Peace and co-authors[3] reported a coverage of 3.1× for the development of the original cherry 6K SNP array and, while Guajardo and co-authors[4] did not target the whole cherry genome, they obtained the equivalent of 0.64× coverage. Thus, a greater coverage might have been needed to identify enough SNPs to ease the focal point strategy. Secondly, Peace et al.[3] and Barac[5] reported that 86% of the cherry reads and 36% of *fruticosa* reads, respectively, did not anchor to the peach WGS[1,2]. SNPs in these reads that did not anchor to the peach WGS were not considered for array development and thus many SNPs present in the genome might have been discarded. Increasing the sequence data pool for development of the +9K add-on would have required new sequencing efforts which was not feasible due to the limited budget to improve the existing array and would have reduced cost-effectiveness of development of the add-on. Use of a cherry WGS was not possible as it was available only after the development of the add-on. Thus, combining available SNPs from multiple studies and using the peach WGS v2 was pursued as an optimal strategy at the time of the development of the add-on.

2. Influence of germplasm on SNP failure rate

SNP success rate might change depending on the germplasm used. In this study, ASSIsT[6] retained 441 SNPs of the original cherry 6K SNP array that had been rejected by ASSIsT in a previous study with the cherry 6K SNP array on sweet cherry germplasm[7]. Similarly, some SNPs retained in this previous study were now rejected. SNPs are not expected to be polymorphic in all germplasm and thus the genotyped germplasm will determine whether SNPs are classified as polymorphic or monomorphic. As such, evaluation of the available focal points and SNP coverage will also change. However, some monomorphic SNPs might have been false positives during SNP detection and thus might never be polymorphic as suggested by Peace et al.[3].

3. Possible reasons for the failure of SNPs found by GBS on P. fruticosa individuals

The *P. fruticosa* germplasm used for SNP discovery might have been different from the ancestral *fruticosa* germplasm of sour cherry used for breeding. In that case, many polymorphisms present in sour cherry would not be present in the genotyped *P. fruticosa* germplasm and vice versa. There is also a chance that some of the detected SNPs were not SNPs within *P. fruticosa* but rather SNPs between *Prunus* species as some *P. fruticosa* individuals have been shown to actually be hybrids[8,9]. However, *P. fruticosa* individuals for SNP detection were chosen carefully to avoid the presence of such hybrids. Polymorphism in the used *P. fruticosa* germplasm might also have only arisen after hybridization with *P.*

avium gave rise to sour cherry. In contrast, *fruticosa* SNPs identified by Peace and et al.[3] were obtained by resequencing direct ancestors of the sour cherry germplasm used here to validate the new 9000 SNPs. Thus, these SNPs represent the currently genotyped germplasm much better. Differences in genome sequence between the *P. fruticosa* germplasm and the sweet and sour cherry genomes might also explain the high failure rates of the *fruticosa* subgenome SNPs identified from Genotyping-by-Sequencing for both sweet cherry and sour cherry. Genotyping *P. fruticosa* individuals with the new array might give more insight into why these SNPs failed. Finally, some SNPs might have failed in the genotyped multi-family germplasm if it was too diverse to distinguish between the different heterozygous groups (i.e., AAAB, AABB, and ABBB). Where SNPs were evaluated in biparental families (results not shown), some SNPs that had failed in the whole germplasm gave reliable results.

References

1. Verde, I. *et al.* The high-quality draft genome of peach (*Prunus persica*) identifies unique patterns of genetic diversity, domestication and genome evolution. *Nature Genetics* **45**, 487–494 (2013).

2. Verde, I. *et al.* The Peach v2.0 release: high-resolution linkage mapping and deep resequencing improve chromosome-scale assembly and contiguity. *BMC Genomics* **18**, 225 (2017).

3. Peace, C. *et al.* Development and evaluation of a genome-wide 6K SNP array for diploid sweet cherry and tetraploid sour cherry. *PLoS ONE* **7**, e48305 (2012).

4. Guajardo, V. *et al.* Construction of high density sweet cherry (*Prunus avium* L.) linkage maps using microsatellite markers and SNPs detected by genotyping-by-sequencing (GBS). *PLoS ONE* **10**, e0127750 (2015).

5. Barac, G. Genotypic and phenotypic diversity and population structure of European ground cherry (*Prunus fruticosa* Pall.). PhD Dissertation, University of Novi Sad., Novi Sad, Serbia (2016).

6. Di Guardo, M. *et al.* ASSIsT: an automatic SNP scoring tool for in- and outbreeding species. *Bioinformatics* **31**, 3873–3874 (2015).

7. Vanderzande, S. *et al.* High-quality, genome-wide SNP genotypic data for pedigreed germplasm of the diploid outbreeding species apple, peach, and sweet cherry through a common workflow. *PLoS ONE* **14**, e0210928 (2019).

8. Macková, L., Vít, P. & Urfus, T. Crop-to-wild hybridization in cherries—Empirical evidence from *Prunus fruticosa*. *Evolutionary Applications* **11**, 1748–1759 (2018).

9. Hrotkó, K., Feng, Y. & Halász, J. Spontaneous hybrids of *Prunus fruticosa* Pall. in Hungary. *Genet Resources and Crop Evolution* (2019) doi:https://doi.org/10.1007/s10722-019-00847-w.
