# Supplementary material for: The cherry 6+9K SNP array: a cost-effective improvement to the cherry 6K SNP array for genetic studies
Source: Sci Rep. 2020 May 6;10:7613. doi: 10.1038/s41598-020-64438-x (PMC7203174; doi:10.1038/s41598-020-64438-x)
Supplement: Supplementary file 3 — Supplementary Information 2 [file 41598_2020_64438_MOESM3_ESM.pdf]

The cherry 6+9K SNP array: a cost-effective improvement to the cherry 6K SNP array for genetic studies

Stijn Vanderzande, Ping Zheng, Lichun Cai, Barac Goran, Ksenija Gasic, Dorrie Main, Amy Iezzoni, Cameron Peace

Supplementary File S2: Clarification of SNP names

All SNP names were kept as originally determined in other studies. The name of SNPs identified from ESTs and organelle SNPs are based on the genes the SNPs were located in[1–3]. Organelle SNP names also include the SNP's position in base pairs. Duplicated SNPs were given a "_2" suffix. All other SNPs from previous studies used the peach whole genome sequence (WGS) v1[4] for their physical position and their names include chromosome (designated as "*X*" below) and position in base pairs (designated as "*YYYY*" below) according to that genome version as follows: SNPs identified by Guajardo et al.[5] were labeled "s*X*_*YYYY*", SNPs identified by Peace et al.[6] were labeled "scaffold_*X*:*YYYY*" for sweet cherry (using lower case s) and "Scaffold_*X*:*YYYY*" for the *fruticosa* subgenome of sour cherry (using upper case S), and SNPs identified by Barac[7] were labeled as "S*X*_*YYYY*". SNPs from the original 6K array were labeled as originally published[6]. Lists of these SNPs using the above nomenclature are in the "SNP name" column (column A) of Supplementary Tables S1-S3.

Although positions of SNPs of the +9K add-on were determined using the peach WGS v2[8] during development and later the sweet cherry WGS v1[9] was used, we kept the original names for the SNPs, i.e., with the peach WGS v1 location in their name (Supplementary Tables S1-S3, column A). This retention maintained consistency in SNP naming between this study and previous studies as well as consistency with names in the original cherry 6K SNP array paper as the SNPs of the original cherry 6K SNP array also had physical positions based on the peach WGS v1 in their names. However, manifest files with updated physical positions for all SNPs based on the peach WGS v2 and the cherry WGS v1 are available from Illumina. These manifest files use the SNP names as listed in Supplementary Tables S1-S3, which can be found in the "Name" column in the GenomeStudio® software.

References

1. Castède, S. *et al.* Mapping of candidate genes involved in bud dormancy and flowering time in sweet cherry (*Prunus avium*). *PLoS ONE* **10**, e0143250 (2015).

2. Pervaiz, T. *et al.* Association between Chloroplast and Mitochondrial DNA sequences in Chinese Prunus genotypes (*Prunus persica*, *Prunus domestica*, and *Prunus avium*). *BMC Plant Biology* **15**, 4 (2015).

3. Distefano, G. *et al.* HRM analysis of chloroplast and mitochondrial DNA revealed additional genetic variability in Prunus. *Scientia Horticulturae* **197**, 124–129 (2015).

4. Verde, I. *et al.* The high-quality draft genome of peach (*Prunus persica*) identifies unique patterns of genetic diversity, domestication and genome evolution. *Nature Genetics* **45**, 487–494 (2013).

5. Guajardo, V. *et al.* Construction of high density sweet cherry (*Prunus avium* L.) linkage maps using microsatellite markers and SNPs detected by genotyping-by-sequencing (GBS). *PLoS ONE* **10**, e0127750 (2015).

6. Peace, C. *et al.* Development and evaluation of a genome-wide 6K SNP array for diploid sweet cherry and tetraploid sour cherry. *PLoS ONE* **7**, e48305 (2012).

7. Barac, G. Genotypic and phenotypic diversity and population structure of European ground cherry (*Prunus fruticosa* Pall.). PhD Dissertation, University of Novi Sad., Novi Sad, Serbia (2016).

8. Verde, I. *et al.* The Peach v2.0 release: high-resolution linkage mapping and deep resequencing improve chromosome-scale assembly and contiguity. *BMC Genomics* **18**, 225 (2017).

9. Shirasawa, K. *et al.* The genome sequence of sweet cherry (*Prunus avium*) for use in genomics-assisted breeding. *DNA Research* **24**, 499–508 (2017).
